# Study of Phase Transformations and Hyperfine Interactions in Fe_3_O_4_ and Fe_3_O_4_@Au Nanoparticles

**DOI:** 10.3390/nano12234121

**Published:** 2022-11-22

**Authors:** Vyacheslav S. Rusakov, Artem L. Kozlovskiy, Maxim S. Fadeev, Kamila B. Egizbek, Assel Nazarova, Kayrat K. Kadyrzhanov, Dmitriy I. Shlimas, Maxim V. Zdorovets

**Affiliations:** 1 Faculty of Physics, M.V. Lomonosov Moscow State University, 119991 Moscow, Russia; 2 Engineering Profile Laboratory, L.N. Gumilyov Eurasian National University, Nur-Sultan 010008, Kazakhstan; 3 Laboratory of Solid State Physics, The Institute of Nuclear Physics, Almaty 050032, Kazakhstan

**Keywords:** Fe_3_O_4_ and Fe_3_O_4_@Au nanoparticles, core-shell structures, Mössbauer spectroscopy, phase transformations, hyperfine interactions

## Abstract

The paper presents the results of a study of iron oxide nanoparticles obtained by chemical coprecipitation, coated (Fe_3_O_4_@Au) and not coated (Fe_3_O_4_) with gold, which were subjected to thermal annealing. To characterize the nanoparticles under study, scanning and transmission electron microscopy, X-ray diffraction, and Mössbauer spectroscopy on ^57^Fe nuclei were used, the combination of which made it possible to establish a sequence of phase transformations, changes in morphological and structural characteristics, as well as parameters of hyperfine interactions. During the studies, it was found that thermal annealing of nanoparticles leads to phase transformation processes in the following sequence: nonstoichiometric magnetite (Fe_3−γ_O_4_) → maghemite (γ-Fe_2_O_3_) → hematite (α-Fe_2_O_3_), followed by structural ordering and coarsening of nanoparticles. It is shown that nanoparticles of nonstoichiometric magnetite with and without gold coating are in the superparamagnetic state with a slow relaxation rate. The magnetic anisotropy energy of nonstoichiometric magnetite is determined as a function of the annealing temperature. An estimate was made of the average size of the region of magnetic ordering of Fe atoms in nonstoichiometric magnetite, which is in good agreement with the data on the average sizes of nanoparticles determined by scanning electron microscopy.

## 1. Introduction

In recent years, among the variety of known nanomaterials, much attention has been paid to iron-containing oxide nanoparticles, the interest in which is due to their wide range of practical applications as well as the potential for their use in almost all fields of science and technology [1,2,3]. Iron-containing nanoparticles also have great potential for catalysis, where they can be used as an absorbent in the catalytic or photocatalytic decomposition of organic dyes or as heavy metal absorbents to extract them from aqueous media, etc. [2,3,4,5,6,7]. An important role is played by iron-containing oxide nanoparticles in the creation of magnetic sensors, as well as in the energy sector, where they are used as the basis for anode materials for lithium-ion batteries [8,9]. In the past few years, areas of application of nanoparticles in medicine have been actively developed, where they can be used as carriers for targeted delivery of drugs, contrast markers for MRI, materials for hyperthermia exposure, etc. At the same time, the emphasis on the use of nanoparticles is shifting more and more in the biomedical direction, which imposes additional requirements on nanostructures related not only to their structural and magnetic properties, but also to resistance to external influences, toxicity, and biological activity [10,11,12,13]. These developments, despite the rather large number of experimental works and reviews, require more and more scientific research in this direction [14,15,16,17]. An analysis of publication activity for such keywords as “magnetic nanoparticles”, “magnetic nanoparticles for biomedical application”, and ““core-shell” magnetic nanoparticles” in the Web of Science Core Collection database showed a growth trend in the number of publications in these areas [18]. The total number of publications over the past two years has more than doubled compared to the same number of publications over the period from 2012 to 2014. At the same time, more than 35% of scientific articles and reviews out of the total number of published works are related to the biomedical application of nanoparticles. Notably, the annual increase in scientific articles and reviews is more than 10%, which indicates an increase in research in this direction.

Separately, it should be noted that one of the most promising areas of research related to iron-containing oxide materials is the creation of core-shell nanostructures or complex nanocomposites [19,20,21,22]. As can be seen from the analysis of publication activity, interest in these types of nanomaterials is increasing every year. The total number of published articles and reviews in this area is more than 30% of the total number of scientific articles related to magnetic nanoparticles.

Interest in core-shell nanostructures is primarily due to the possibilities for their application, which are significantly expanded via shell-coating with noble metals, such as Au and Ag, or any polymer or organosilicon compound on magnetic nanoparticles [23,24,25]. Coatings on magnetic nanoparticles not only increase their resistance to external factors that can lead to corrosion and subsequent degradation, but also increase the possibility of binding various complex compounds or drugs to the surface of nanoparticles [26,27]. Such structures make it possible to create nanocontainers with a magnetic core, due to which it is possible to control nanoparticles using external magnetic fields. In this case, the nonmagnetic shell reduces the effect of agglomeration of magnetic nanoparticles into larger complexes. These properties, as well as a wide variety of other properties and their variations, make core-shell nanoparticles very promising objects for research [28,29,30].

The purpose of this work is to study the effect of thermal annealing of Fe_3_O_4_ and Fe_3_O_4_@Au nanoparticles on changes in their morphology and structural properties, on the kinetics of temperature phase transformations, and on hyperfine interactions of Fe^57^ nuclei. The choice of Fe_3_O_4_ and Fe_3_O_4_@Au nanoparticles as objects of study is due to the prospects for practical application in microelectronics, batteries, catalysis, purification of aqueous media, and biomedicine [31,32,33]. At the same time, the previously proposed method for modifying Fe_3_O_4_ nanoparticles by depositing a gold shell on them makes it possible to obtain core-shell structures [34]. However, to date, little is known about the effect of coating with a gold shell on the change in structural parameters and the dynamics of phase transformations during thermal action on nanoparticles, as a result of which, phase transformation processes are initiated in iron-containing oxide nanoparticles. Much attention is paid to the analysis of the parameters of magnetic hyperfine interactions, as well as their connection with data on changes in structural parameters and phase transformations. It should be noted that the use of Mössbauer spectroscopy, in contrast to classical X-ray methods, makes it possible to answer several questions related to the phase analysis and nonstoichiometry of iron-containing oxide phases in nanoparticles during their thermal annealing.

## 2. Experimental Part

The initial nanoparticles were synthesized using the method of chemical coprecipitation from 2M FeCl_2_ and 1M FeCl_3_ salts with the addition of 50 mL of ammonium hydroxide (NH_4_OH) to the solution. The deposition of the gold shell was carried out in two stages, including the dispersion of nanoparticles in a solution of citric acid (with a concentration of 0.1 g/mL) followed by treatment in a solution of gold chloride and sodium citrate. A detailed description of the procedure for the synthesis of iron-containing nanoparticles, as well as the deposition of a gold shell on them, is presented in [34].

To initialize the processes of phase transformations in nanoparticles, the method of thermal annealing in an air atmosphere was applied. The samples were annealed in a SNOL muffle furnace in a temperature range of 100–800 °C with a step of 100 °C for 5 h, and followed by cooling the samples to room temperature for 10–24 h, depending on the annealing temperature.

The morphological features of the synthesized nanoparticles were studied using high-resolution scanning electron microscopy (SEM) and high-resolution transmission electron microscopy (TEM). For these studies, a Jeol 7500F scanning electron microscope (Jeol, Tokyo, Japan) and a Jeol JEM1400 Plus transmission electron microscope (Jeol, Tokyo, Japan) were used. SEM and TEM images were processed using ImageJ software V.2.0 (Github Inc, San Francisco, CA, USA).

The analysis of structural changes and phase transformations as a result of thermal annealing was carried out using the X-ray diffraction (XRD) method implemented on D8 Advance ECO (Bruker, Berlin, Germany) and MiniFlex 600 (Rigaku Corporation, Tokyo, Japan) diffractometers. The diffraction patterns were taken in the Bragg–Brentano geometry, in the angular range 2θ = 25–85°, with a step of 0.03°. The diffraction patterns were processed using the SmartLab Studio II software and the ICDD PDF-2 database.

Investigations of hyperfine interactions of ^57^Fe nuclei were carried out at room temperature by Mössbauer spectroscopy (MS) on an MS1104Em spectrometer (Research Institute of Physics, Rostov State University. Rostov-on-Don, Russia) operating in the constant acceleration mode with a triangular change in the Doppler velocity of the source relative to the absorber. The ^57^Co nuclei in the Rh matrix acted as a source of resonant γ-quanta. The Mössbauer spectrometer was calibrated at room temperature using an α-Fe reference absorber. The obtained Mössbauer spectra were analyzed using the SpectrRelax software (MSU, Moscow, Russia) [35].

## 3. Results and Discussion

**Figure 1:**
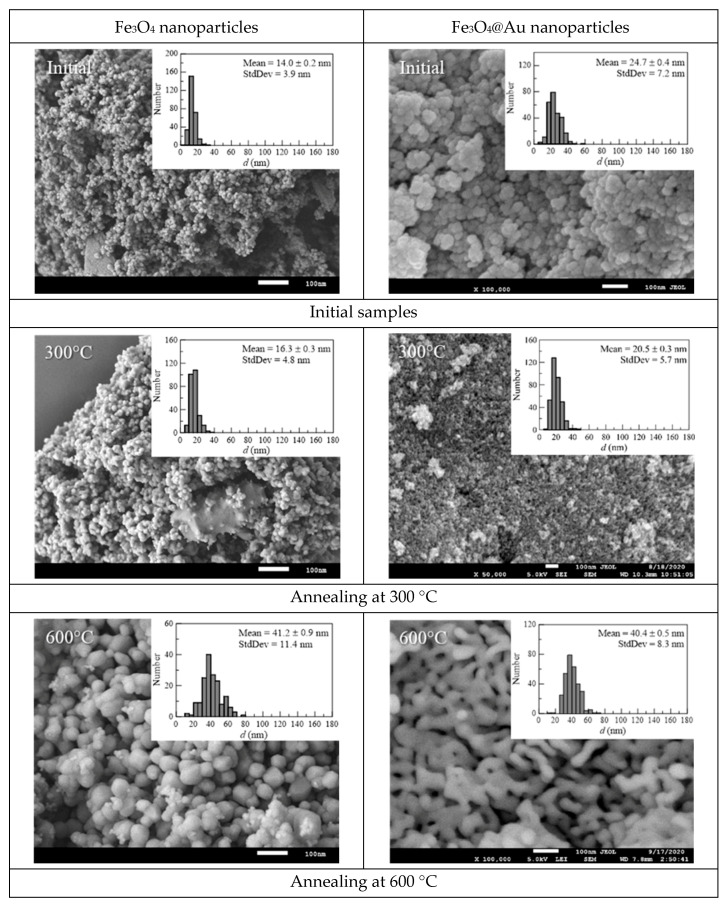

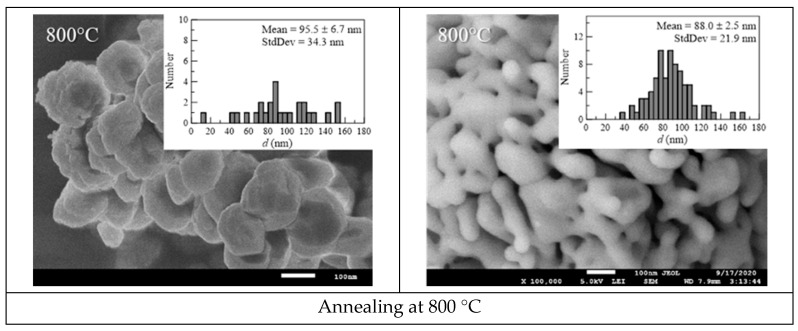
SEM images and size distributions of the studied Initial (initial samples) and Annealed (annealing at *t*_ann_) Fe_3_O_4_ and Fe_3_O_4_@Au nanoparticles.

The results of the study of the morphological features of synthesized and annealed Fe_3_O_4_ and Fe_3_O_4_@Au nanoparticles, obtained using SEM, are shown in Figure 1. Analysis of SEM images of nanoparticles and their size distributions shows that the shape of nanoparticles at low annealing temperatures is close to spherical, both in the case of Fe_3_O_4_ nanoparticles, and Fe_3_O_4_@Au nanoparticles.

**Figure 2:**
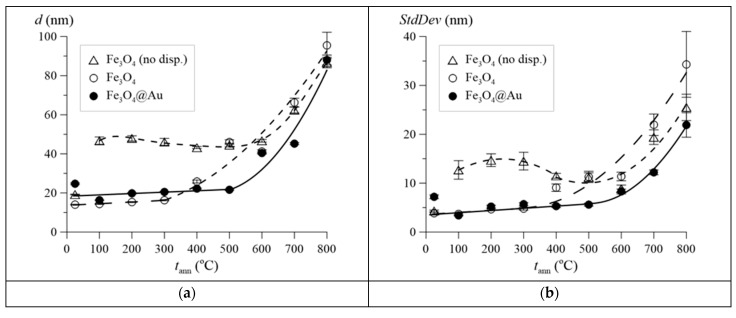
Results of evaluation of average nanoparticle sizes (**a**) and standard deviation of nanoparticle size distribution (**b**) as a function of annealing temperature *t*_ann._

Figure 2 shows the results of estimating the average size of nanoparticles obtained from the SEM image analysis data, depending on the annealing temperature *t*_ann_. For Fe_3_O_4_@Au nanoparticles after synthesis and subsequent annealing at temperatures of 100–300 °C, the average particle sizes exceed the average sizes of Fe_3_O_4_ nanoparticles by 4–6 nm, which indicates the presence of a shell with a thickness of 2–3 nm.

At temperatures of 100–400 °C, undispersed Fe_3_O_4_ nanoparticles with an average size of ~15–20 nm stick together, forming agglomerates of particles with an average size of ~45 nm, which are easily dispersed under ultrasonic treatment. A further increase in the annealing temperature leads to sticking and combination of the initial dispersed and non-dispersed nanoparticles with the formation of larger nanoparticles, up to ~90 nm at *t*_ann_ = 800 °C.

It should be noted that the process of particle association begins for dispersed Fe_3_O_4_ nanoparticles at ~400 °C, and for Fe_3_O_4_@Au nanoparticles, at a higher temperature of ~550 °C (Figure 2a). Such association of nanoparticles occurs primarily due to particles with the most probable and largest sizes, as evidenced by the increase in the width of the size distributions of formed nanoparticles with increasing annealing temperature (see Figure 2b). At the same time, the average size and width of the size distribution at the same annealing temperature are noticeably larger for Fe_3_O_4_ nanoparticles than for Fe_3_O_4_@Au nanoparticles, for which the presence of a shell prevents their association.

**Figure 3:**
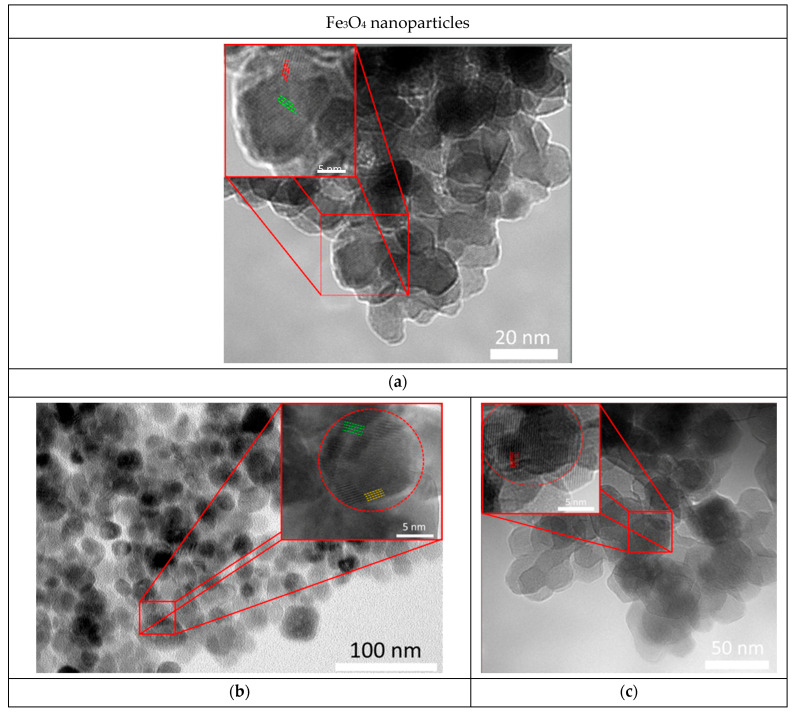
(**a**) TEM images of studied initial (Initial) and annealed (Annealing) (**a**) Fe_3_O_4_ nanoparticles at temperatures of 400 °C (**b**) and 600 °C (**c**).

Figure 3 shows TEM images (including high resolution) of the investigated Initial and Annealed at temperatures of 400 °C and 600 °C Fe_3_O_4_ nanoparticles. According to the presented data, unannealed Fe_3_O_4_ nanoparticles consist of several crystallites (regions of structural ordering). At the same time, the interplanar distances of the main part of the crystallites are characteristic of magnetite with an unknown stoichiometry degree (*d*(Fe_3_O_4_)_(022)_ = 3.00 ± 0.13 Å). For nanoparticles annealed at a temperature of 400 °C, there are two different orientations of parallel atomic planes with different interplanar distances, presumably corresponding to nonstoichiometric magnetite (*d*(Fe_3_O_4_)_(111)_ = 4.41 ± 0.23 Å) and hematite (*d*(α-Fe_2_O_3_)_(012)_ = 3.59 ± 0.16Å). At the same time, for nanoparticles annealed at a temperature of 600 °C, only one system of parallel atomic planes with an interplanar spacing characteristic of hematite is observed.

**Figure 4:**
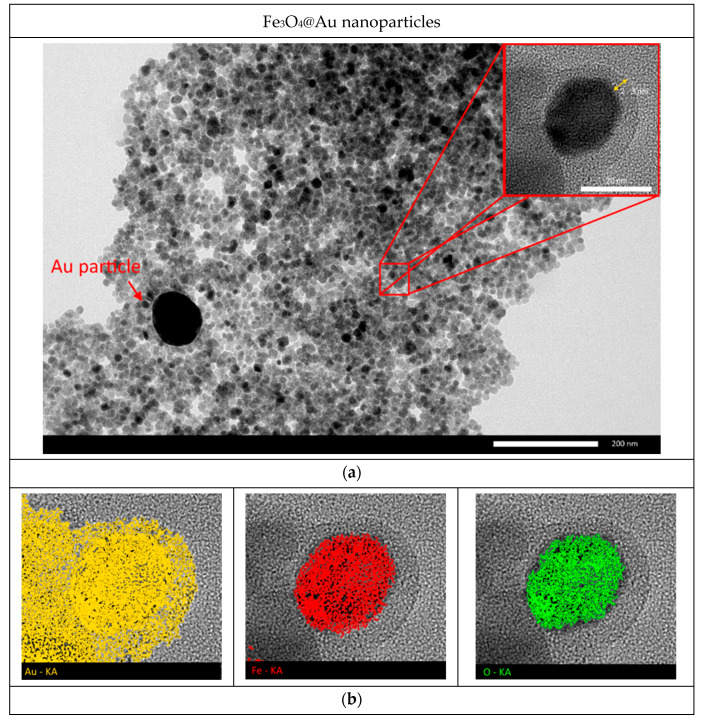
(**a**) TEM image of Fe_3_O_4_@Au nanoparticles (inset shows high-resolution TEM image of Fe_3_O_4_@Au nanoparticles); (**b**) mapping results of Fe_3_O_4_@Au nanoparticles.

A TEM image of Fe_3_O_4_@Au nanoparticles and high-resolution image of one Fe_3_O_4_@Au nanoparticle, as well as mapping results of this nanoparticle, are shown in Figure 4. Analysis of the obtained TEM images (Figure 4a) shows that the studied Fe_3_O_4_@Au nanoparticles have a core-shell structure, where the core is a nanoparticle consisting of iron oxide (dark central area in the high-resolution image), and its lighter shell, according to the mapping results, is a shell of gold (see Figure 4b). The shell thickness was estimated to be 3–5 nm, which is in good agreement with the values obtained by scanning electron microscopy (SEM) as a result of comparing the average sizes of Fe_3_O_4_ and Fe_3_O_4_@Au nanoparticles.

The TEM image of Fe_3_O_4_@Au nanoparticles (Figure 4a) shows a single large particle, which is very different from the rest of the nanoparticles. According to the result of elemental analysis, this particle is a gold particle, the presence of which may be associated with the process of gold agglomeration during synthesis.

**Figure 5:**
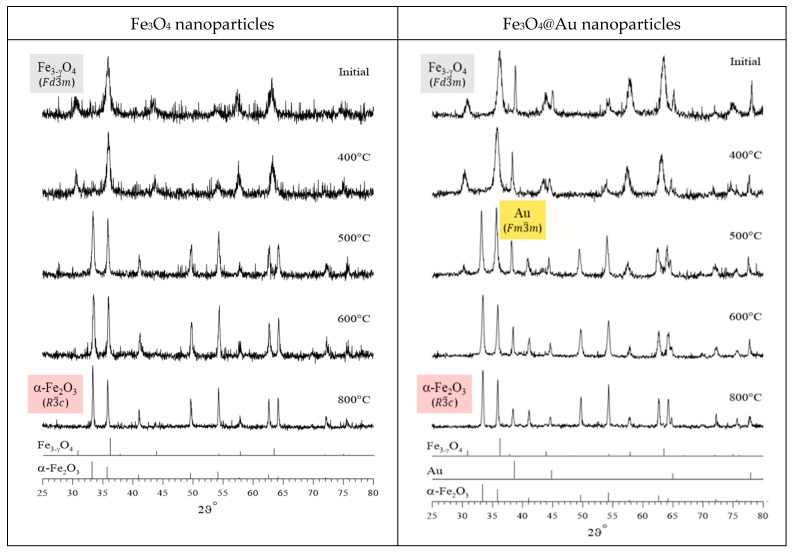
X-ray diffraction patterns of initial and annealed at temperatures of 400 °C, 500 °C, 600 °C, and 800 °C Fe_3_O_4_ and Fe_3_O_4_@Au nanoparticles.

The results of X-ray diffraction, showing the dynamics of phase transformations in the studied nanoparticles depending on the annealing temperature, are shown in Figure 5. An analysis of the obtained data made it possible to establish that the initial nanoparticles are nanoparticles of nonstoichiometric magnetite Fe_3−γ_O_4_ with an inverse spinel structure (sp. gr. $Fd\bar{3}m$). Along with an increase in the annealing temperature of nanoparticles, magnetite is oxidized, as a result of which the diffraction patterns reveal reflections characteristic of the α-Fe_2_O_3_ hematite phase with a rhombohedral structure (sp. gr. $R\bar{3}c$); the contribution of such reflections increases with an increase in the annealing temperature (Figure 5). As annealing temperature increases, a decrease in the widths of the diffraction reflections of the nonstoichiometric magnetite and hematite phases is observed, which indicates the ordering of the crystal structures and an increase in the sizes of the regions of structural ordering (coherence lengths) for both phases. In the case of Fe_3_O_4_@Au nanoparticles, the diffraction patterns contain reflections (Figure 5) characteristic of gold (Au) nanoparticles with a face-centered cubic crystal lattice (sp. gr. $Fm\bar{3}m$), the presence of which was established using transmission electron microscopy (Figure 4).

**Figure 6:**
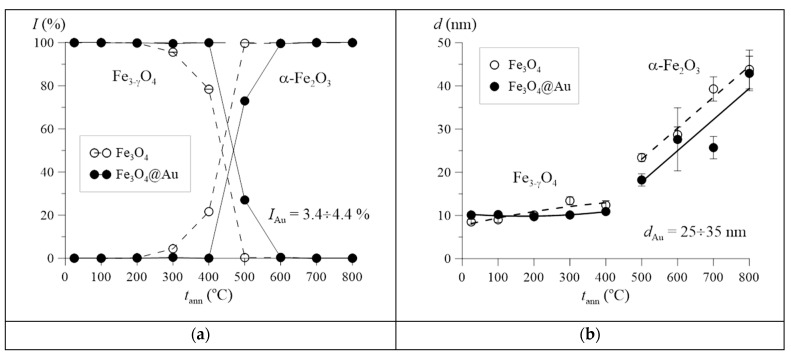
Dependences of relative intensities *I* of diffraction patterns of nonstoichiometric magnetite Fe_3−γ_O_4_ and hematite α-Fe_2_O_3_ (**a**), as well as the average size *d* of the regions of structural ordering of nanoparticles of nonstoichiometric magnetite Fe_3−γ_O_4_ and hematite α-Fe_2_O_3_ (**b**) on the annealing temperature *t*_ann._

Figure 6a shows the dependences of the relative intensities of the diffraction patterns of the established phases on the annealing temperature *t*_ann_. For Fe_3_O_4_ nanoparticles, already at *t*_ann_ = 300 °C, along with nonstoichiometric Fe_3−γ_O_4_ magnetite, the appearance of the α-Fe_2_O_3_ hematite phase (*I* ~ 3%) is fixed, which becomes dominant (*I* ~ 100%) at annealing temperatures *t*_ann_ ≥ 500 °C. In the case of Fe_3_O_4_@Au nanoparticles, the oxidation of nonstoichiometric magnetite (Fe_3−γ_O_4_) and its transformation into hematite (α-Fe_2_O_3_) occurs at *t*_ann_ > 450 °C. At the same time, for Fe_3_O_4_@Au, the contribution of the diffraction pattern of Au nanoparticles (*I* ~ 4%) and the unit cell parameter of Au (*a* ~ 4.082 Å) remain unchanged during annealing, which indicates the absence of decomposition (peeling) of the shell and the formation of Au nanoparticles, as well as the appearance of a substitution phase or introductions.

Depending on the annealing temperature, Figure 6b shows the average values *d* of the sizes of regions of structural ordering (coherence lengths/crystallite sizes) of nanoparticles, determined using the Scherrer formula. As can be seen from the data presented, up to an annealing temperature of 400 °C, only a slight increase in the coherence length is observed, which is associated mainly with the oxidation and ordering of the crystal structure of nonstoichiometric magnetite. In this case, the association of nanoparticles, and as a result a sharp increase in the coherence length, occurs at higher temperatures, when they are mainly hematite particles. At the same time, as we see in Figure 6b, for Fe_3_O_4_@Au nanoparticles, the Au shell makes it difficult for them to combine; the size of coated nanoparticles is, on average, ~5 nm smaller than that of Fe_3_O_4_ nanoparticles after combining.

**Figure 7:**
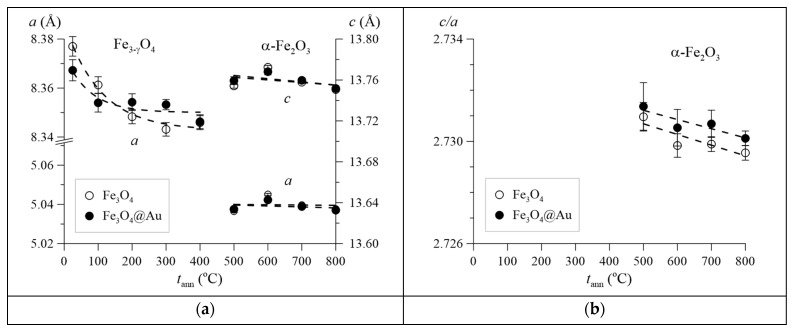
Unit cell parameters for nonstoichiometric magnetite Fe_3−γ_O_4_ and hematite α-Fe_2_O_3_ (**a**), as well as ratios of unit cell parameters *a*/*c* of hematite α-Fe_2_O_3_ (**b**) depending on the annealing temperature *t*_ann._

As a result of the processing of X-ray diffraction patterns by the Rietveld method for the studied Fe_3_O_4_ and Fe_3_O_4_@Au nanoparticles, the unit cell parameters of the crystal lattice for nonstoichiometric magnetite Fe_3−γ_O_4_ and hematite α-Fe_2_O_3_ were determined depending on the annealing temperature *t*_ann_ (Figure 7a). In the case of nonstoichiometric magnetite Fe_3−γ_O_4_, the presence of which is typical for *t*_ann_ ≤ 400 °C, a noticeable decrease in the unit cell parameter is observed with an increase in the annealing temperature, which indicates an increase in the degree of its nonstoichiometry γ [36,37]. For hematite, the unit cell parameters slightly decrease with the annealing temperature. At the same time, a slight decrease in the ratio of unit cell parameters *c/a* indicates the improvement of its crystal structure (Figure 7b) [38]. Since in the case of nanoparticles of nonstoichiometric magnetite, it is not possible to accurately determine the degree of its nonstoichiometry γ by X-ray diffraction, then, including for this purpose, the method of Mössbauer spectroscopy was applied.

**Figure 8:**
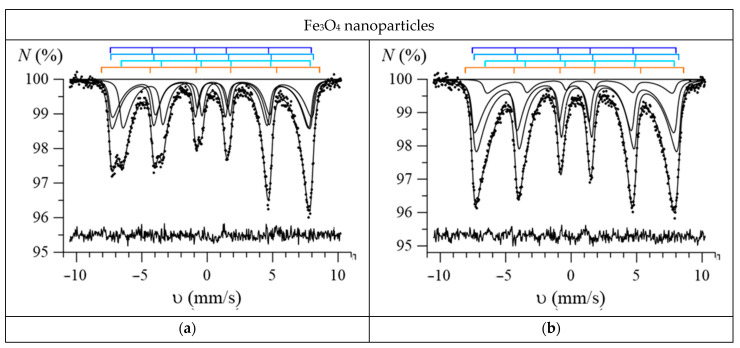

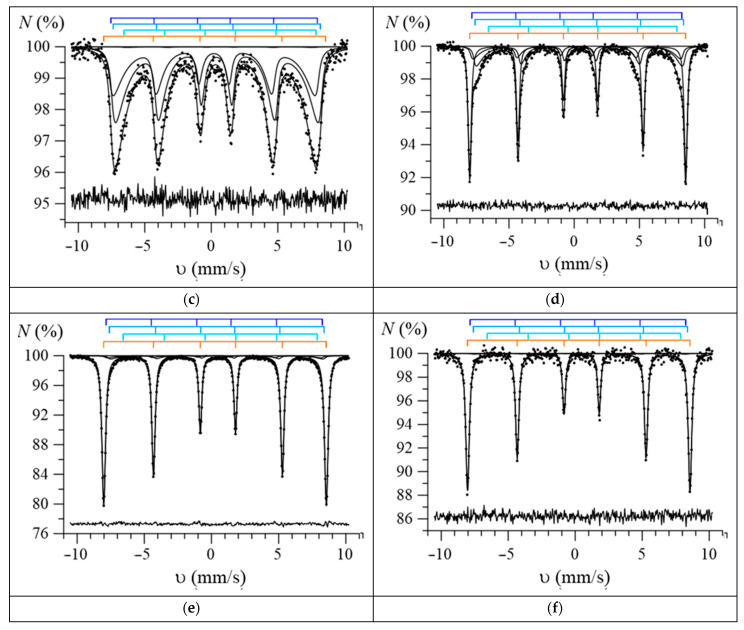
Mössbauer spectra of the initial (**a**) and annealed at temperatures of 100 °C (**b**), 200 °C (**c**), 400 °C (**d**), 600 °C (**e**), 800 °C (**f**) Fe_3_O_4_ nanoparticles (on each spectrum the positions of the resonance lines of the subspectra used for model fitting are shown; the lines corresponding to magnetite are shown in blue, and the lines corresponding to hematite are shown in orange).

**Figure 9:**
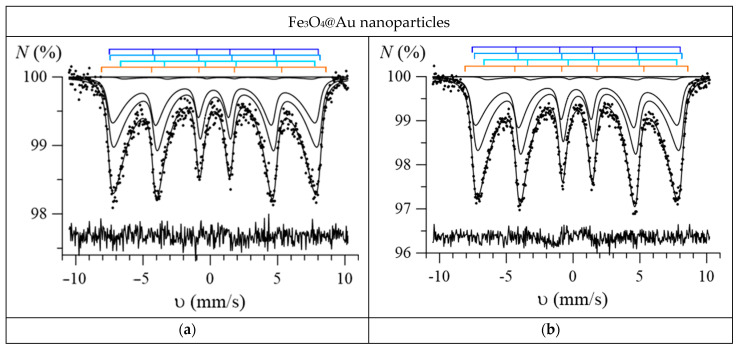

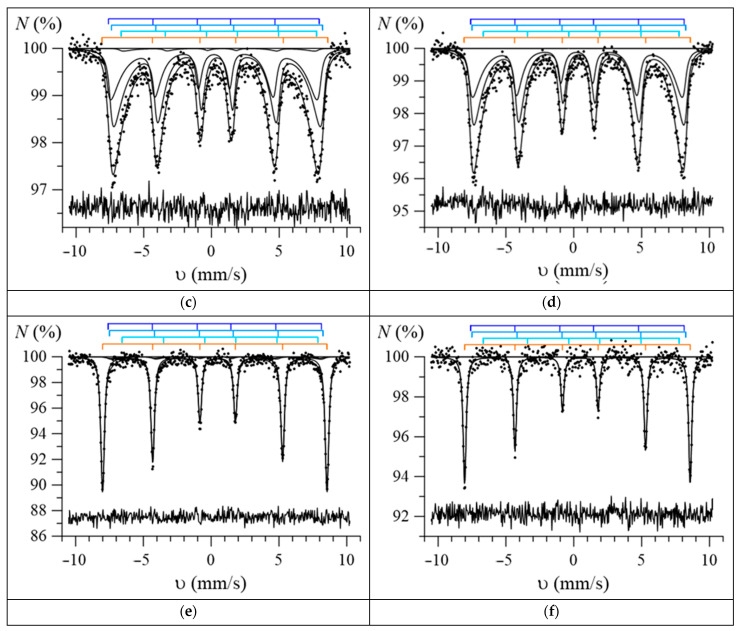
Mössbauer spectra of the initial (**a**) and annealed at temperatures of 100 °C (**b**), 200 °C (**c**), 400 °C (**d**), 600 °C (**e**), 800 °C (**f**) Fe_3_O_4_@Au nanoparticles (on each spectrum shows the positions of the resonance lines of the subspectra used for model fitting; the lines corresponding to magnetite are shown in blue, and the lines corresponding to hematite are shown in orange).

Figure 8 and Figure 9 show the most characteristic Mössbauer spectra of the studied Fe_3_O_4_ and Fe_3_O_4_@Au nanoparticles. As can be seen, these spectra, especially for nanoparticles annealed at low temperatures (100–300 °C), are poorly resolved and show signs of the relaxation behavior of nanoparticles. Therefore, for the model fitting and interpretation of these spectra, a priori information about the properties of nanoparticles obtained using electron microscopy and X-ray diffraction methods was used and reasonable physical assumptions were made.

**Figure 10:**
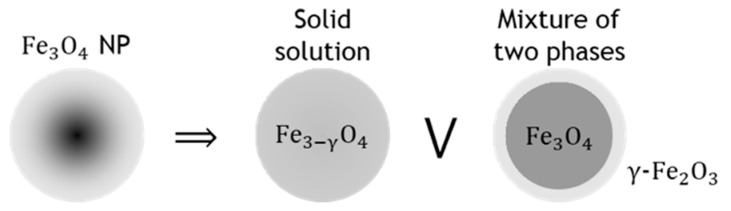
Diagram of limiting options for representing a magnetite nanoparticle with a spatially heterogeneous nonstoichiometry degree.

In the general case, a magnetite nanoparticle can be represented as a particle with a gradient change in the degree of magnetite nonstoichiometry. The limiting options for representing such a particle are either a particle of homogeneous composition with an average value of the nonstoichiometry degree γ, i.e., a solid solution of magnetite Fe_3_O_4_ and maghemite γ-Fe_2_O_3_, or a mixture of magnetite and maghemite, for example, in the center and on the surface of the particle, respectively (see Figure 10).

In the presence of fast electron exchange, regardless of the type of representation of the crystal chemical formula of the studied oxide, it contains trivalent iron ions in the tetrahedral (A) position $\left({\mathrm{Fe}}_{A}^{3+}\right)$, trivalent iron ions in the octahedral (B) position $\left({\mathrm{Fe}}_{B}^{3+}\right)$, as well as ions with intermediate valence in the octahedral position $({\mathrm{Fe}}_{B}^{2.5+}$), participating in the Verwey mechanism of electron exchange [39,40].

The crystal chemical formula in the presence of a fast electron exchange between Fe atoms in the case of a solid solution of magnetite and maghemite (nonstoichiometric magnetite Fe_3−γ_O_4_) is written as

(1) $${\mathrm{Fe}}_{3-\gamma }O_{4}={\left({\mathrm{Fe}}^{3+}\right)}_{A}{\left[{\mathrm{Fe}}_{2\left(1-3\gamma \right)}^{2.5+}{\mathrm{Fe}}_{5\gamma }^{3+}{☐}_{\gamma }\right]}_{B}O_{4}^{2-},$$

and in the case of a mixture of magnetite and maghemite phases as

(2) $$\left(1-b\right)\cdot {\left({\mathrm{Fe}}^{3+}\right)}_{A}{\left[{\mathrm{Fe}}_{2}^{2.5+}\right]}_{B}O_{4}^{2-}+b\cdot {\left({\mathrm{Fe}}^{3+}\right)}_{A}{\left[{\mathrm{Fe}}_{\frac{5}{3}}^{3+}{☐}_{\frac{1}{3}}\right]}_{B}O_{4}^{2-},$$

where γ is the number of vacancies of Fe atoms (☐) per formula unit (degree of nonstoichiometry), 0 ≤ *b* ≤ 1 is the molar concentration of maghemite, while *b* = 3γ.

According to crystal chemical Formulas (1) and (2), it is possible to determine the molar concentration of maghemite (b) and the number of vacancies of iron atoms per formula unit (*γ*) using the intensity ratios of the subspectra corresponding to different iron ions, taking into account the known probability ratio of the Mössbauer effect for Fe atoms in octahedral and tetrahedral positions (*f*_B_*/f*_A_ = 0.94 ± 0.02 [41]):

(3) $$b=3\gamma =\frac{3}{5}\cdot \frac{f_{A}}{f_{B}}\cdot \frac{I\left({\mathrm{Fe}}_{B}^{3+}\right)}{I\left({\mathrm{Fe}}_{A}^{3+}\right)}=1-\frac{1}{2}\cdot \frac{f_{A}}{f_{B}}\cdot \frac{I\left({\mathrm{Fe}}_{B}^{2.5+}\right)}{I({\mathrm{Fe}}_{A}^{3+})}.$$

Since the obtained Mössbauer spectra are characteristic of the relaxation behavior of the studied nanoparticles, when fitting them, it is necessary to use the model of multilevel superparamagnetic relaxation [42]. Some of its main parameters are the relaxation rate (*R*) and the ratio of the magnetic anisotropy energy ($E_{\mathrm{ma}}=K_{\mathrm{eff}}V$) to the thermal energy ($k_{B}T$):

(4) $$\alpha =\frac{E_{\mathrm{ma}}}{k_{B}T}=\frac{K_{\mathrm{eff}}V}{k_{B}T}.$$

As can be seen, using the relaxation model, it is possible to estimate the volumes (*V*), and hence the characteristic sizes of the magnetic ordering regions, if we use the values of the effective magnetic anisotropy coefficients *K*_eff_ of nanoparticles at different annealing temperatures. The values of the coefficients *K*_eff_ were estimated using the literature data on the magnetic anisotropy coefficients for stoichiometric magnetite (Fe_3_O_4_) and maghemite (γ-Fe_2_O_3_), as well as the values of the molar concentration of maghemite *b* obtained from the model fitting of the Mössbauer spectra [34].

Using SpectrRelax, a program for processing and analyzing Mössbauer spectra [35], a model was implemented for deciphering the Mössbauer spectra of iron oxides in the form of nanoparticles of a mixture of magnetite (Fe_3_O_4_) and maghemite (γ-Fe_2_O_3_) or nanoparticles of nonstoichiometric magnetite (Fe_3–γ_O_4_) in the presence of fast electron exchange, taking into account multilevel superparamagnetic relaxation for Fe atoms in various structural and charge states [34]. Thus, when fitting the spectra of nanoparticles, we used a model consisting of three interconnected relaxation subspectra of nonstoichiometric magnetite (corresponding to ${\mathrm{Fe}}_{A}^{3+}$, ${\mathrm{Fe}}_{B}^{3+}$ and ${\mathrm{Fe}}_{B}^{2.5+}$), to which an independent Zeeman sextet corresponding to hematite was added. The use of this model for fitting the spectra made it possible to establish changes in the following characteristics with the annealing temperature: the molar concentration of maghemite *b*, the magnetite nonstoichiometry degree *γ*, the magnetic anisotropy energy *E*_ma_, the magnetic anisotropy coefficient *K*_eff_, and the size of the region of magnetic ordering of iron atoms *d* in nonstoichiometric magnetite. As can be seen in Figure 8 and Figure 9, all experimental spectra are well-described within the model used: the normalized chi-squared values for Fe_3_O_4_ nanoparticles range from 0.96 to 1.36, and for Fe_3_O_4_@Au nanoparticles, from 0.97 to 1.20. It should be noted that slow superparamagnetic relaxation was observed for all nanoparticles when the relaxation time was noticeably longer (by about 2 orders of magnitude) than the lifetime of the ^57^Fe nucleus in the excited state (the relaxation rate R was noticeably smaller than the natural width of the excited state level).

**Figure 11:**
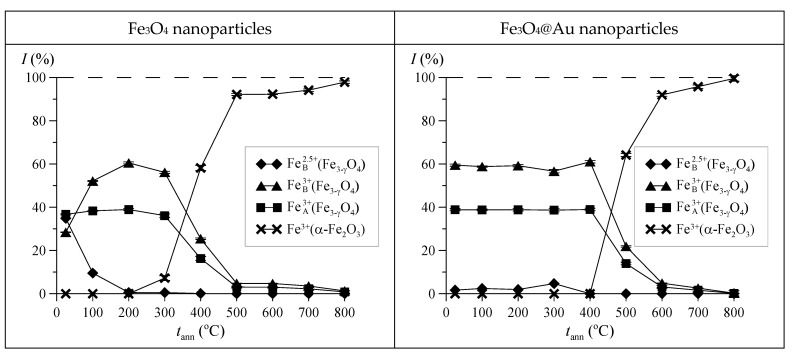
Relative intensities of subspectra corresponding to different states of Fe atoms in Fe_3_O_4_ and Fe_3_O_4_@Au nanoparticles, depending on the annealing temperature *t*_ann._

Figure 11 shows the relative intensities of subspectra corresponding to different states of Fe atoms in Fe_3_O_4_ and Fe_3_O_4_@Au nanoparticles, depending on the annealing temperature. An analysis of the obtained data showed that at annealing temperatures below 300 °C, Fe_3_O_4_ nanoparticles are non-stoichiometric magnetite Fe_3−γ_O_4_, while with an increase in the annealing temperature in the octahedral position (B) of the inverse spinel structure, oxidation of iron atoms occurs—an increase in the relative number of Fe^3+^ ions due to a decrease in the number of Fe^2.5+^ ions. Above the annealing temperature of 300 °C, nonstoichiometric magnetite in Fe_3_O_4_ nanoparticles transforms into hematite (α-Fe_2_O_3_).

**Figure 12:**
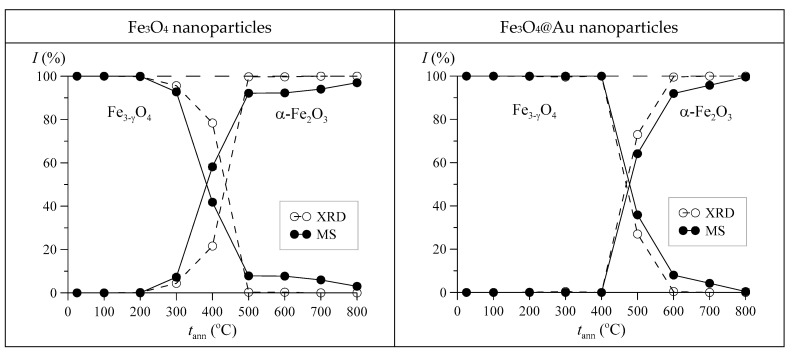
Dependences of the relative contributions to the Mössbauer spectrum (MS) and X-ray diffraction pattern (XRD) of nonstoichiometric magnetite and hematite in Fe_3_O_4_ and Fe_3_O_4_@Au nanoparticles on the annealing temperature *t*_ann._

For Fe_3_O_4_@Au nanoparticles, the same sequence of phase changes is observed, only at higher annealing temperatures, by approximately 150 °C. It should be noted that the dependences of the relative contributions to the Mössbauer spectrum (MS) and X-ray diffraction pattern (XRD) of magnetite and hematite in nanoparticles on the annealing temperature are in good agreement with each other (see Figure 12).

**Figure 13:**
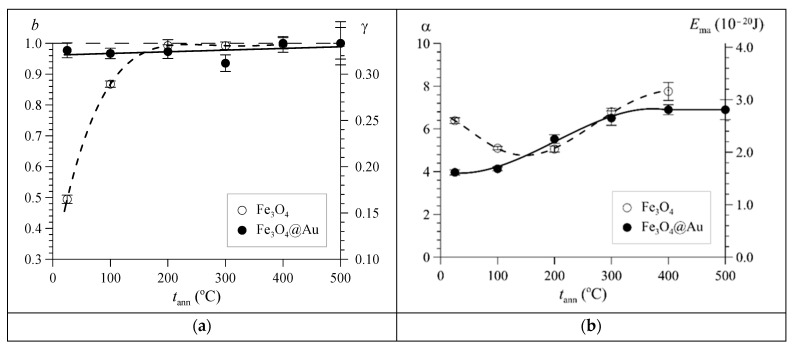

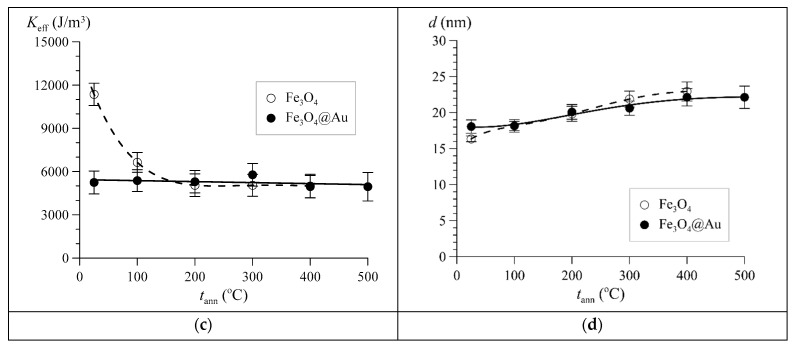
Dependences on the annealing temperature *t*_ann_ of the molar concentration of maghemite *b* and the nonstoichiometry degree γ (**a**), the parameter of the multilevel superparamagnetic relaxation model α and the magnetic anisotropy energy *E*_ma_ (**b**), the magnetic anisotropy coefficient *K*_eff_ (**c**), and the average size of the magnetic ordering region *d* (**d**) for nonstoichiometric magnetite in Fe_3_O_4_ and Fe_3_O_4_@Au nanoparticles.

Figure 13 shows data on non-stoichiometric magnetite depending on the annealing temperature, obtained as a result of model fitting of the Mössbauer spectra of the studied nanoparticles. For the initial Fe_3_O_4_ nanoparticles, the molar concentration of maghemite γ-Fe_2_O_3_ was *b* = 0.49 ± 0.01, and the magnetite nonstoichiometry degree was γ = 0.165 ± 0.004. Nonstoichiometric magnetite in the initial Fe_3_O_4_ nanoparticles is completely oxidized to maghemite γ-Fe_2_O_3_ at *t*_ann_ ≥ 200 °C. The core of the initial Fe_3_O_4_@Au nanoparticles is mainly maghemite (*b =* 0.98 ± 0.02, γ = 0.326 ± 0.008) (Figure 13a), since the oxidation of magnetite in it occurs already in the synthesis process.

The parameter of the multilevel superparamagnetic relaxation model α, equal to the ratio of the magnetic anisotropy energy to the thermal energy (4), and the magnetic anisotropy energy *E*_ma_ as a function of the annealing temperature are shown in Figure 13b. It can be seen that for Fe_3_O_4_@Au nanoparticles, the energy *E*_ma_ increases monotonically, while for Fe_3_O_4_ nanoparticles, a rather “convoluted” dependence is observed at low annealing temperatures. This behavior of the dependence *E*_ma_(*t*_ann_) can be explained if we take into account the oxidation of nanoparticles not coated with gold. In accordance with the data on the molar concentration of maghemite *b*, the effective coefficients of magnetic anisotropy of nanoparticles *K*_eff_ were determined, the results of which are shown in Figure 13c. As can be seen, for Fe_3_O_4_@Au nanoparticles, the *K*_eff_ coefficient practically remains unchanged, while for Fe_3_O_4_ nanoparticles it sharply decreases with temperature in accordance with the oxidation of nonstoichiometric magnetite to maghemite (Figure 13a). If this value differs for magnetic nanoparticles from the bulk samples of magnetite presented in [43,44,45], this may be due to size factors, as well as structural disordering of the initial magnetite nanoparticles, which also leads to a change in the magnetic parameters.

Using the results of estimates of the effective coefficient of magnetic anisotropy, in accordance with (4), the average sizes of the magnetic ordering regions of Fe atoms in nonstoichiometric magnetite for Fe_3_O_4_ and Fe_3_O_4_@Au nanoparticles were calculated; they are presented in Figure 13d. The assessment of the “magnetic ordering region” for uncoated Fe_3_O_4_ samples annealed at 500–800 °C and for coated Fe_3_O_4_@Au samples at 600–800 °C was not carried out due to the small contribution of Fe_3−γ_O_4_ to the experimental spectrum (see Figure 9e,f), making it impossible to reliably find the values of the relaxation model parameter α (see Formula (4) and Figure 9e,f). It is evident that with an increase in the annealing temperature, the average sizes of these regions gradually increase both due to the oxidation of nanoparticles and due to their structural and magnetic ordering. Note that the average size of the magnetic ordering region increases from 16.4 ± 0.4 to 18.1 ± 0.9 nm as a result of gold coating of the initial nanoparticles, which is associated with oxidation during gold coating. At the same time, the obtained values of the average size of the magnetic ordering region are in good agreement with the data of scanning electron microscopy (Figure 2a).

As a result of model fitting of the Mössbauer spectra of Fe_3_O_4_ and Fe_3_O_4_@Au nanoparticles subjected to thermal annealing, the values of hyperfine parameters of subspectra (hyperfine magnetic fields *H*_n_, isomer shifts δ, and quadrupole shifts ɛ of resonance lines) were obtained, which made it possible to unambiguously identify subspectra and analyze their dependences on the annealing temperature.

**Figure 14:**
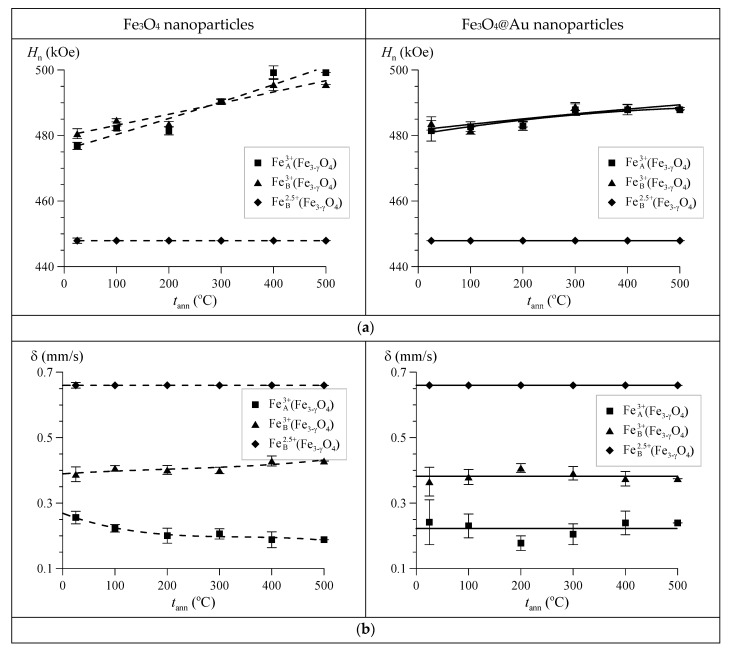

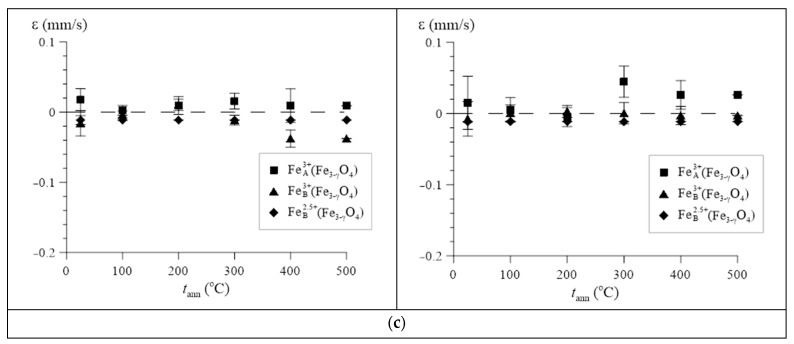
Dependences on the annealing temperature *t*_ann_ of the hyperfine magnetic field *H*_n_ (**a**), the isomer shift δ (**b**), and the quadrupole shift ɛ (**c**) for nonstoichiometric magnetite in Fe_3_O_4_ and Fe_3_O_4_@Au nanoparticles.

Figure 14, which demonstrates the hyperfine parameters of the subspectra of nonstoichiometric magnetite ${\mathrm{Fe}}_{3-\gamma }O_{4}$, shows that the isomer shifts δ for trivalent iron ions vary in the ranges of 0.19–0.26 mm/s and 0.39–0.43 mm/s (Figure 14b), which is typical for tetrahedral (A) and octahedral (B) oxygen environments in the ${\mathrm{Fe}}_{3-\gamma }O_{4}$ structure.

The hyperfine magnetic fields *H*_n_ on the ^57^Fe nuclei for trivalent iron ions in the tetra- and octahedral positions are quite close ($△H_{n}=H_{n}^{B}-H_{n}^{A}\leq 4$ kOe) and, unlike the other hyperfine parameters for these ions, at *t*_ann_ ≤ 400 °C, they noticeably increase with increasing annealing temperature, which indicates both improvement of the structure as well as an increase in the magnetite nonstoichiometry degree. This is also evidenced by the observed small changes in the isomer shifts of the subspectra of nonstoichiometric magnetite (Figure 14b). As for the quadrupole shifts, their values turned out to be close to zero (Figure 14c).

**Figure 15:**
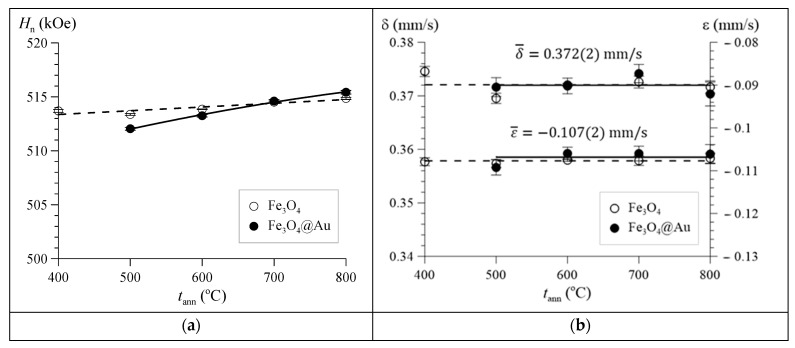
Dependences on the annealing temperature *t*_ann_ of the hyperfine magnetic field *H*_n_ (**a**), the isomer shift δ, and the quadrupole shift ɛ (**b**) for hematite α-Fe_2_O_3_ in Fe_3_O_4_ and Fe_3_O_4_@Au nanoparticles.

Dependences of the hyperfine parameters of the subspectrum of hematite α-Fe_2_O_3_ in Fe_3_O_4_ and Fe_3_O_4_@Au nanoparticles on the annealing temperature are shown in Figure 15.

It can be seen that the isomer shift and the quadrupole shift of the resonance lines practically do not change, and the hyperfine magnetic field somewhat increases with an increase in the annealing temperature, approaching a value corresponding to the literature data for pure bulk hematite. Thus, we can conclude that with an increase in the annealing temperature, the crystal and magnetic structure of hematite is improved.

## 4. Conclusions

Fe_3_O_4_ and Fe_3_O_4_@Au nanoparticles were studied using scanning and transmission electron microscopy, X-ray diffraction, and Mössbauer spectroscopy, as a result of which a sequence of phase transformations and a change in the morphology and structure of nanoparticles depending on the annealing temperature were established. Using the Mössbauer spectroscopy method, the nonstoichiometry degree γ of nonstoichiometric magnetite Fe_3−γ_O_4_ in the studied nanoparticles was determined. It has been established that the initial Fe_3_O_4_ nanoparticles with sizes of 10–18 nm and a shape close to spherical are nonstoichiometric magnetite nanoparticles with a stoichiometry degree γ = 0.165 ± 0.004. Initial Fe_3_O_4_@Au nanoparticles have a core-shell structure with a 2–3 nm thick gold shell and a core consisting mainly of extremely oxidized magnetite—maghemite γ-Fe_2_O_3_ (γ = 0.326 ± 0.008). At low annealing temperatures *t*_ann_ (100–400 °C), Fe_3_O_4_ nanoparticles stick together, forming particle agglomerates with an average size of ~45 nm that are easily dispersed under ultrasonic treatment. Higher annealing temperatures lead to sticking and coalescence of the initial dispersed and non-dispersed nanoparticles with the formation of larger nanoparticles (up to ~90 nm at 800 °C) consisting of hematite α-Fe_2_O_3_. The presence of a gold shell in Fe_3_O_4_@Au nanoparticles prevents their association. In this case, the transformation of nonstoichiometric magnetite into hematite for Fe_3_O_4_@Au nanoparticles occurs at annealing temperatures ~150 °C higher than for Fe_3_O_4_ nanoparticles (at ~300 °C).

It has been shown by Mössbauer spectroscopy that nonstoichiometric magnetite nanoparticles with and without gold coating are in the superparamagnetic state with a slow relaxation rate compared to the reciprocal lifetime of the ^57^Fe core in the excited state. Depending on the annealing temperature, the energy of the magnetic anisotropy of nonstoichiometric magnetite was determined and an estimate was made of the average size of the region of magnetic ordering of Fe atoms in nonstoichiometric magnetite, which is in good agreement with the data on the sizes of nanoparticles determined by scanning electron microscopy.

## Data Availability

Not applicable.
